# Short-term associations of air pollution and meteorological variables on the incidence and severity of COVID-19 in Madrid (Spain): a time series study

**DOI:** 10.1186/s12302-021-00548-1

**Published:** 2021-09-06

**Authors:** Cristina Linares, Fernando Belda, José Antonio López-Bueno, M. Yolanda Luna, Gerardo Sánchez-Martínez, Beatriz Hervella, Dante Culqui, Julio Díaz

**Affiliations:** 1 National School of Public Health, Carlos III Institute of Health, Avda. Monforte de Lemos 5, 28029 Madrid, Spain; 2 State Meteorological Agency (AEMET), Madrid, Spain; 3 The UNEP DTU Partnership, Copenhagen, Denmark

**Keywords:** COVID-19, Temperature, Air pollution, Hospital admissions, Incidence rate

## Abstract

**Background:**

There are studies that analyze the role of meteorological variables on the incidence and severity of COVID-19, and others that explore the role played by air pollutants, but currently there are very few studies that analyze the impact of both effects together. This is the aim of the current study. We analyzed data corresponding to the period from February 1 to May 31, 2020 for the City of Madrid. As meteorological variables, maximum daily temperature (Tmax) in ºC and mean daily absolute humidity (AH) in g/m^3^ were used corresponding to the mean values recorded by all Spanish Meteorological Agency (AEMET) observatories in the Madrid region. Atmospheric pollutant data for PM_10_ and NO_2_ in µg/m^3^ for the Madrid region were provided by the Spanish Environmental Ministry (MITECO). Daily incidence, daily hospital admissions per 100.000 inhabitants, daily ICU admissions and daily death rates per million inhabitants were used as dependent variables. These data were provided by the ISCIII Spanish National Epidemiology Center. Generalized linear models with Poisson link were performed between the dependent and independent variables, controlling for seasonality, trend and the autoregressive nature of the series.

**Results:**

The results of the single-variable models showed a negative association between Tmax and all of the dependent variables considered, except in the case of deaths, in which lower temperatures were associated with higher rates. AH also showed the same behavior with the COVID-19 variables analyzed and with the lags, similar to those obtained with Tmax. In terms of atmospheric pollutants PM_10_ and NO_2,_ both showed a positive association with the dependent variables. Only PM_10_ was associated with the death rate. Associations were established between lags 12 and 21 for PM_10_ and between 0 and 28 for NO_2_, indicating a short-term association of NO_2_ with the disease. In the two-variable models, the role of NO_2_ was predominant compared to PM_10_.

**Conclusions:**

The results of this study indicate that the environmental variables analyzed are related to the incidence and severity of COVID-19 in the Community of Madrid. In general, low temperatures and low humidity in the atmosphere affect the spread of the virus. Air pollution, especially NO_2,_ is associated with a higher incidence and severity of the disease. The impact that these environmental factors are small (in terms of relative risk) and by themselves cannot explain the behavior of the incidence and severity of COVID-19.

## Introduction

There is currently no clear scientific evidence that environmental factors such as temperature and humidity affect the spread of the new SARS-CoV-2 virus or the slowing of transmission. What is known is that the influence of environmental factors is lesser compared to the public health measures implemented to contain the virus [1]. The reasons why many respiratory viruses are typical in winter in temperate regions is related to different factors that contribute to this seasonal phenomenon: lower humidity conditions during the winter period, different human activity (greater use of closed spaces in winter) and to the host’s immune system (generally more susceptible in winter) [2]. Different epidemiological studies have been published at the population level documenting different velocities of spread between different geographical zones with varying climatological factors. The data from one study [3] suggest that there is an association between a country’s latitude and mortality rates of COVID-19. This gradient can be observed within a country, such as Italy, in which the North of the country is more affected than the South [4]. In other countries, this is not the case, however. There is no observed association in the United States between deaths and cases by latitude of individual states [5]. Some authors have suggested that there could be a pattern determined by temperature and by humidity, with a decrease in the intensity of transmission associated with an increase in temperature and relative humidity [6].

On the other hand, studies have analyzed the role of local level air pollution, which seems to be an environmental factor that can aggravate the COVID-19 disease process and increase its severity. However, the possible effect on the spread of the virus is still in question, especially if particulate matter (PM) pollutants are capable of serving as a viable means of transport for the new SARS-CoV-2 virus [7]. Another more widespread hypothesis is focused on the greater cardio-respiratory vulnerability presented by those people who are habitually exposed to high levels of air pollution in cities. According to the World Health Organization (WHO), 1 out of 7 patients with COVID-19 suffer from respiratory problems and other severe complications [8]. Up until now, the factors associated with mortality due to COVID-19 include sex (greater risk among men), age (greater risk in those over age 65) and the presence of co-morbidities such as hypertension, diabetes, and cardiovascular and cerebrovascular diseases. Vascular inflammation, myocarditis and cardiac arrhythmias have also been documented in relation to this new disease. All of these illnesses overlap in large part with the causes of mortality related to the exposure to particulate matter and their impact on health.

While many publications have addressed the influence of environmental factors on the incidence and severity of COVID-19, the majority of these studies use different methodological approximations. There are studies that report simple correlations between the health variables related to COVID-19 and air pollution [9–11] or in the case of meteorological variables, simple correlations with temperature and humidity [12]. There are also studies, though lesser in number, that analyze the two types of variables jointly regarding the incidence of COVID-19 [13]. In terms of modeling, studies have generally been published that report the results of single-variable models for room temperature or absolute humidity [14, 15], with similar results in that they reflect the negative relationship between these two environmental variables and those related to COVID-19 [16].

While it is true that the majority of published studies show methodological deficiencies both terms of the time series analyzed and the control variables (in some cases nonexistent they do provide plausible results and hypotheses that deserve to be considered, especially if the development and implementation of plans to decrease pollution in cities can be a tool in addressing the spread and severity of the SARS-CoV-2 virus.

In this study, we analyze the combination of the association of meteorological variables of temperature and absolute humidity and the registered concentrations of pollutants such as PM_10_ and NO_2_ on the incidence rates and severity of COVID-19 registered in the Community of Madrid during the period of the state of alarm in Spain.

## Methods

### Dependent and independent variables

The dependent variables were calculated based on the number of positive cases of COVID-19. Cases diagnosed as positive for COVID-19 were defined based on positive PCR test results in 99.74% of the data. The remaining cases were diagnosed based on presentation of symptoms compatible with the disease.

Cases defined as such refer to the daily cases that occurred in the Community of Madrid during the time period between February 1, 2020 and May 31, 2020. The state of alarm and subsequent confinement of the population was decreed by the Spanish State March 14, with the application of measures for restricted movements and social interaction [17]. This state of alarm remained in place until June 21 [18].

We analyzed data corresponding to the number of cases diagnosed as positive for COVID-19 in different categories: number of cases, number of emergency hospital admissions, number of intensive care unit (ICU) admissions and the number of deaths due to COVID-19. Data were provided by the National Center for Epidemiology at the Carlos III Institute. The data for this period related to the population of the Community of Madrid were supplied by the National Statistics Institute (INE). Based on these data, we calculated the following: COVID-19 incidence rate per 100,000 inhabitants; rate of emergency hospital admissions for COVID-19 per 100,000 inhabitants; rate of ICU hospital admissions for COVID-19 per 1,000,000 inhabitants and COVID-19 death rate per 1,000,000 inhabitants.

The independent variables consisted of meteorological data and air pollution data.

Meteorological data were the values of daily maximum temperature (Tmax), minimum temperature (Tmin), and average temperature (Tmed) in Celsius and daily average relative humidity (RH) in percent.

The values of average absolute daily humidity (AH) in g/m^3^ were obtained based on average relative humidity and average daily humidity [19].

These values constitute the average value of the observations corresponding to the AEMET stations located in the Community of Madrid and were provided by the State Meteorological Agency (AEMET).

The air pollution data were the average daily values of the concentrations of PM_10_ and NO_2_ in μg/m^3^, obtained by using the average of the values measured in the stations located in the Community of Madrid. These data were provided by the Ministry of Ecological Transition and Demographic Change (MITECO).

Fourteen-day average values were calculated based on the average daily values for these independent variables.

### Methodology of analysis

First, time lags with p-values below 0.05 were detected that existed between the different variables for the rates of COVID cases described. Cross-correlation functions (CCF) were established between the residuals of the prewhitened series for these variables. Prewhitening was carried out through ARIMA single-variable modeling. Knowing time lags was necessary to analyze the temporality of case detection, hospital admission and admission to the ICU, and death.

Generalized linear models with Poisson link (GLM) were carried out between the dependent (positive COVID rates) and independent (environmental) variables. In these models we controlled for the series trend and seasonality for 120, 90, 60 and 30 days, and the autoregressive nature of the series. Also, we have controlled for weekly seasonality.

$$Log\left(y\right)=a+ {\beta }_{1}{n1}_{i}+{\beta }_{2}{seas}_{i}+{\beta }_{3}{env}_{i}+{\beta }_{4}{lag(X,g)}_{i}+{\varepsilon }_{i},$$

where *y* represents each of the dependent variables previously commented, *a* is the intercept. β_n_ represents the coefficient of each of the n variables. *n1* is trend, *seas* represents each of the variables used to control seasonality, *env* represents each of the environmental variables, *lag*(X, g) represents the lagged variable of order g, in which X has been replaced by the dependent variable and environmental variables and ε the represents residuals of the model; each of them at the i observation.

GLM were carried out between each dependent variable and the average daily values of the independent variables, and later with the 14-day average values. This single-variable modeling allowed us to determine which of the daily temperatures we worked with presented better associations with dependent variables. In this way, time lags were established that produced associations with *p*-values below 0.05 between the dependent variables and the independent variables (*p* < 0.005).

The range of lag days considered in the analysis is from 0 to 28 days to take into account the time that took place between the occurrence of symptoms and worsening of symptoms and arrival at the hospital [20, 21] and the lag times between incidence, admission in the ICU and death in Spain [22]. A weekly distributed lag model has been used. In a first step, the lags have been introduced corresponding to the independent variables lagged from 0 to 7 days. In a second step, the lags corresponding to 8–14 days have been introduced, keeping the variables lagged that were statically significant in the first step, and so on up to 28 days to complete the range of lag days considered in the analysis. The lagged variables will appear as follows, “short-term" for lags between 0 and 7 days; “medium term” for lags between 7 and 14 days and "long term” for the remaining lags.

Later, two-variable models were carried out, including the air pollution variables and the meteorological variables. Finally, all variables models were carried out between the entire dependent and independent variables introducing the control variables described. Based on the absolute values of the estimators, relative risks (RR) were calculated in the form RR = e^β^ with β as the absolute value of the estimator obtained in the Poisson modeling. A negative coefficient in the estimator indicates that an increase in the independent variable is associated with a decrease in the dependent variable. The RR is calculated by an increase of 1 µg/m3 of PM_10_ and NO_2_; 1 ºC in the maximum temperature (Tmax) and 1 g/m^3^ in the absolute humidity (AH) value.

We used a back-stepwise process for variable selection, and statistical significance was set at a p-value of p < 0.05. Over- or under-dispersion have been controlled.

## Results

Table 1 shows the descriptive statistics of the dependent variables (COVID-19 rates) and independent variables analyzed during the study period. It is worth highlighting that, in terms of average values, 47.1% of detected cases were admitted to the hospital, 2.9% were admitted to the ICU, and 11.9% died.

**Table 1:** Descriptive statistics of the COVID-19 rate variables and independent variables analyzed during the period 02-01-2020 to 05-31-2020

	Maximum	Minimum	Mean	Std. deviation
Incidence rate*	42.53	0.03	8.68	11.12
Hospital admissions rate*	25.71	0.03	4.16	6.45
Intensive care unit admissions rate**	15.76	0.00	2.54	3.98
Mortality rate **	49.22	0.00	10.30	12.91
Daily maximum temperature (Tmax) (°C)	30.4	8.4	17.6	5.5
Absolute humidity AH (g/m^3^)	11.9	1.9	7.3	1.7
PM_10_ (μg/m^3^)	85.1	5.1	15.8	12.2
NO_2_ (μg/m^3^)	57.3	2.5	18.8	13.6

^*^Cases per 100,000 inhabitants. **Cases per million inhabitants

**Fig. 1:**
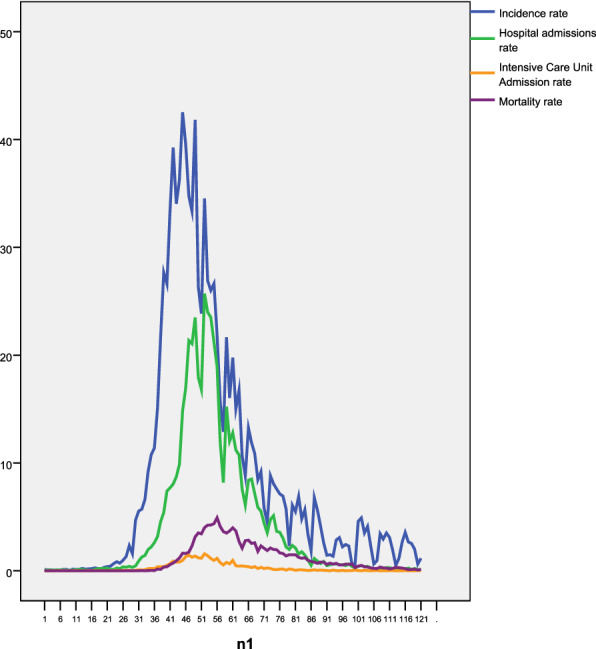
Temporal evolution of incidence rate; hospital admissions rate; intensive care unit admission rate, and mortality rate from 01 February 2020 to 31 May 2020 (n1). All in cases per 100.000 inhabitants

**Fig. 2:**
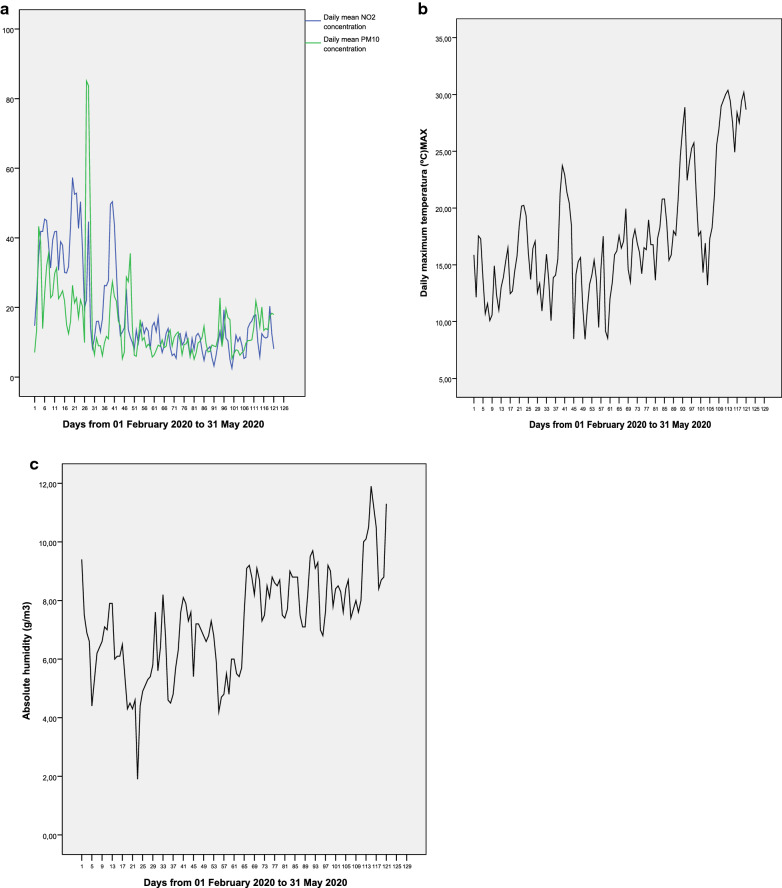
Temporal evolution of: **a** daily mean concentration NO_2_ and PM_10_ (μg/m^3^); **b** daily maximum temperature (°C) and **c** daily mean absolute humidity (g/m^3^)

Figure 1 shows the temporal evolution of the dependent variables, and Fig. 2 shows that of the independent variables. In terms of the dependent variables, we observed a time lag between the incidence rate and the hospital admission rate, as well as between the ICU admission rate and the death rate. The evolution of the concentrations of NO_2_ and PM_10_ in Fig. 2 shows, in both cases, a clear trend of decline. Furthermore, in the case of PM_10_, there are periodic increases in the average daily concentrations of the pollutant. In contrast, in the case of maximum daily temperatures (Fig. 2b), there is a clear trend of increase, which is also the case for absolute average daily humidity (Fig. 2c).

**Fig. 3:**
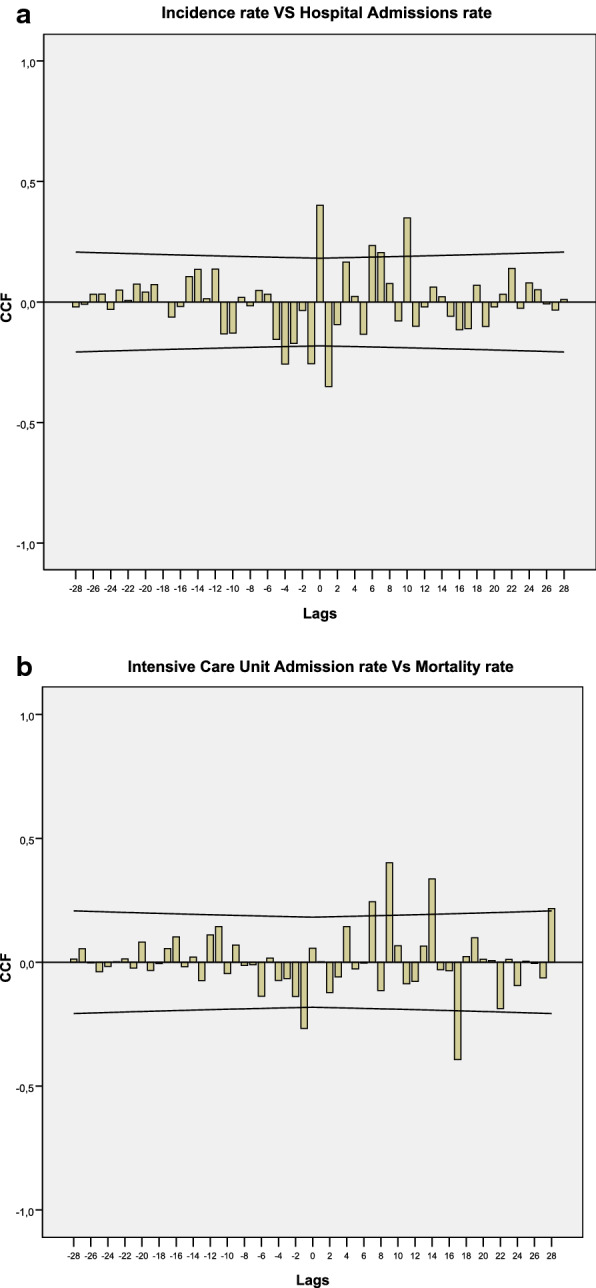
Cross-correlation function (CCF): **a** incidence rate vs hospital admissions rate; **b** intensive care unit admission rate vs mortality rate

In order to establish a lag between the series of incidence and that of admissions, and the series of ICU admissions and deaths, we carried out crossed correlation functions between the different series, as described in the methodology. The lags with p-values below 0.05 between incidence and admissions were produced for lags 0, 6, 7 and 10 (at short and medium term), between incidence and ICU admissions for lags 0 and 7 (at short term), and between incidence and deaths for lags 7, 11 and 13 (at medium term). There were associations with p-values below 0.05 between admissions and ICU admissions for lags 0 and 9 (at short and medium term), and finally, there were associations with p-values below 0.05 between ICU admissions and deaths for the lags 7, 9, 14 and 28 (at medium and long term). For example, Fig. 3 shows some of these crossed correlation functions.

Table 2 shows the Pearson’s correlations coefficients between independent variables.

**Table 2:** Pearson’s bivariate correlations between daily mean concentrations of NO_2_ and PM_10_; daily maximum temperature (Tmax) and absolute humidity (AH)

	NO_2_	PM_10_	Tmax	AH
NO_2_	1	0.519**	− 0.143	− 0.527**
PM_10_	0.519**	1	0.019	− 0.182*
Tmax	− 0.143	0.019	1	0.569**
AH	− 0.527**	− 0.182	0.569**	1

^**^ Significance *p* < 0.001; * significance *p* < 0.05

Table 3 shows the principal results in terms of the associations found between the different COVID-19 rates and the daily values of the independent variables in terms of the lags in which there were associations with p-values before 0.05, both in the single-variable and two-variable models. It also shows the relative risks obtained for the all variables models that include Tmax and AH as well as NO_2_ and PM_10_.

**Table 3:** Lags in which associations are established between the daily values of the independent variables and the analyzed COVID-19 variables

		PM_10_ (µg/m^3^)	NO_2_ (µg/m^3^)	Daily maximum temperature Tmax (°C)	Absolute humidity AH (g/m^3^)
Incidence rate	Single variable	12	0/14/21	7/14	7/16/18/23
	Two variables	*Without effect*	0/14/21	14	16/18/23
	All variables	*Without effect*	0/14	14	18/23
Hospital admissions rate	Single variable	20	5/19	7/14/27	4/16/20/25/29
	Two variables	*Without effect*	5/19	7/14	16/20
	All variables	18	*Without effect*	7/14	16/20
Intensive care unit admissions rate	Single variable	14/19	0/21/28	11	15/21/23
	Two variables	*Without effect*	0/21/28	11	15/21
	All variables	*Without effect*	21/28	*Without* *effect*	15/21
Mortality rate	Single variable	21	*Without effect*	*Without* *effect*	*Without* *effect*

Regarding PM_10_ there was an association with p-value below 0.05, with positive coefficients for the four COVID-19 rates analyzed. These associations were established between the lags of 12 and 21 (at medium and long term). On the other hand, the average daily concentrations of NO_2_ also showed an association with p-value below 0.05, with positive coefficients for the COVID-19 variables, except for the death rate. In addition to the similar lags obtained for PM_10_, NO_2_ showed a short-term association (lags 0 and 5) that was not found for PM_10_. Once two-variable models were carried out that included average daily concentrations of PM_10_ and NO_2_ the association of NO_2_ was predominant in all of the models.

In contrast to the pollutant variables, the meteorological variables analyzed showed associations with p-values below 0.05 with negative coefficients both for maximum daily temperature (Tmax) and absolute humidity (AH). In general Tmax was associated with all of the COVID variables, both in the medium-term (lags 7–14) and in the long-term (lag 27), but less so than AH. There was no association with the mortality rate. In two-variable models the impact of AH was predominant over Tmax.

Also Table 3 shows the results of the all variables models with respect to daily values. The results agree with what has already been mentioned: the value of NO_2_ was predominant over PM_10_, except for the admissions rate; there was a short-term association of NO_2_ on incidence; and an association of AH and Tmax.

Finally, Table 4 shows the behavior of the 14-day average values of the independent variables compared to the COVID variables analyzed. These results were very similar to those described for the daily values, though some differences are worth highlighting. First, there was an association between average values and mortality, both for PM_10_ and NO_2_, which was not the case for Tmax and AH. The models with all variables showed the predominance of the associations related to NO_2_ compared to those of PM_10_ as well as those of AH compared to Tmax already described.

**Table 4:** Lags in which associations are established between the averaged over 14 days-values of the independent variables and the analyzed COVID-19 variables

		PM_10_ (µg/m^3^)	NO_2_ (µg/m^3^)	Daily maximum temperature Tmax (ºC)	Absolute humidity AH (g/m^3^)
Incidence rate	Single variable	11	13	23	14
	Two variables	11	13	*Without effect*	14
	All variables	*Without effect*	13	*Without effect*	14
Hospital admissions rate	Single variable	14	28	7/20	4/20/29
	Two variables	*Without effect*	28	*Without effect*	4/20/29
	All variables	*Without effect*	28	*Without effect*	4/20/29
Intensive care unit admission rate	Single variable	20	21/28	21	20
	Two variables	*Without effect*	21/28	*Without effect*	7/20
	All variables	*Without effect*	21/28	*Without effect*	*Without effect*
Mortality rate	Single variable	21	28	*Without effect*	*Without effect*

Table 5 shows the relative risks corresponding to final models with all the independent variables, both daily values and averaged values with p-values below 0.05.

**Table 5:** Relative risks corresponding to final models with all the independent variables

	Daily values	Averaged values (0–14 days)
Incidence rate	NO_2_ RR: 1.02 (1.00, 1.04) Tmax RR: 1.05 (1.02, 1.07) AH RR: 1.19 (1.16, 1.22)	NO_2_ RR: 1.04 (1.02, 1.06) AH RR: 1.37 (1.16, 1.58)
Hospital admissions rate	PM_10_ RR: 1.01 (1.00, 1.02) Tmax RR: 1.14 (1.10, 1.17) AH RR: 1.23 (1.20, 1.27)	NO_2_ RR: 1.05 (1.02, 1.08) AH RR: 3.12 (2.09, 4.14)
Intensive care unit admissions rate	NO_2_ RR: 1.02 (1.00, 1.05) AH RR: 1.20 (1.00, 1.39)	NO_2_ RR: 1.10 (1.06, 1.14)

The RR is calculated by an increase of 1 µg/m3 of PM_10_ and NO_2_; 1 °C in the maximum temperature (Tmax) and 1 g/m^3^ in the absolute humidity (AH) value

## Discussion

Given the analysis of many different factors, this section is structured into different parts to facilitate reading and comprehension.

### Variables for COVID-19 rates and detected temporality

During the time this analysis was carried out, PCR tests for detecting positive cases of COVID-19 were only used for those people who presented symptoms compatible with the disease. According to a later prevalence study, SARS-CoV-2 in Spain (ENE-COVID) [23], “One in three infections seems to be asymptomatic, while a substantial number of symptomatic cases remained untested”. Thus, the confirmation of cases during the study period analyzed was carried out in large part among those who presented symptoms with a certain level of severity. This would explain the high percentage of admissions (47.1 percent of all detected cases) and of deaths in Madrid in relation to the incidence of the disease (11.9%) and in relation to data from other European and Spanish cities [24], which was almost triple that established by the WHO, which indicated a percentage of around 4% for deaths and diagnoses [8].

This severity of diagnosed cases would also explain the existence of the association in lag 0 between the incidence and rates of hospital admission and even ICU admission. That is to say, these were people who were diagnosed with the disease and were admitted due to its severity to the hospital and even the ICU on the same day of diagnosis. The lags found between the incidence rate and hospital admissions in lags 6, 7 and 10 (at short and medium term) are also compatible with the time that took place between the occurrence of symptoms and worsening of symptoms and arrival at the hospital [20, 21]. On the other hand, the lag times found between incidence, admission in the ICU and death were similar to those expected in the evolution of COVID-19 in Spain [22].

### The relationship with air pollution variables

The time evolution of the concentrations of the pollutants analyzed, PM_10_ and NO_2_, clearly showed a trend of decline, which could be explained by the restrictions on mobility that took place in Madrid after the declaration of the state of alarm on March 14. The decline observed in the concentrations of the pollutants in the last week of the study, compared to the first, were 34.5% for PM_10_ and 66.8% for NO_2_, which shows the marked anthropic origin of the NO_2_ in Madrid and the important natural origin component (including processes of resuspension) of PM_10_ concentrations [25]. “Peaks” in PM_10_ can be observed in Fig. 2, related to the advection of particulate matter of Saharan origin during these dates [26]. The declines in the concentrations of PM_10_ and NO_2_ are similar to those found in other cities in Spain during the confinement [27, 28].

Table 3 shows the existence of an association between average daily concentrations of PM_10_ and NO_2_ and the COVID-19 incidence rate, the rate of hospital admissions and ICU admissions. There is even an association with *p*-value below 0.05 detected between PM_10_ and the death rate.

There are two biological mechanisms that could explain the existence of these associations [29]. On one hand, is clear that air pollution affects human health [30]. On the other hand, Pothirat et al. [31] investigated the association between daily average seasonal air pollutants and daily mortality of hospitalized patients and community dwellers, as well as emergency and hospitalization visits for serious respiratory, cardiovascular, and cerebrovascular diseases. It was found that air pollutants were associated with higher mortality of the hospitalized patients and community dwellers, with varying effects on severe acute respiratory, cardiovascular, and cerebrovascular diseases. In relation to the age of the individuals who are affected by outdoor air pollution—with particular attention to the respiratory system—those of elderly ages are one of the most sensitive groups [32–34]. That is to say that air pollution worsens the same type of pathology in the same vulnerable age groups impacted more severely by SARS-Cov2 [22].

The other mechanism is based on the fact that air pollution weakens the immune system in the short term. There is growing evidence that pollution can induce oxidative stress, resulting in the production of free radicals, which in turn, may damage the respiratory system, reducing the resistance to viral and bacterial infections [35]. Air pollutants could influence the immune system and affect its ability to limit the spread of infectious agents like the Respiratory Syncytial Virus (RSV) [36, 37]. On the other hand, Zhao et al. [38] has established that short-term exposure to PM_2.5_ could act on the balance of inflammatory M1 and anti-inflammatory M2 macrophage polarizations, a fact that might be involved in air pollution-induced immune disorders and diseases.

Furthermore, in the case of PM concentrations, there is another possible mechanism related to the transmission of the virus. According to a study carried out in Lombardia [7], traces of RNA of SARS-CoV-2 were found in samples of PM measured both in industrial and urban settings in Bergamo. The authors suggest that the aerosol particles that contain the virus of between 0,1 and 1 µm can travel further when they group together with pollutant particles of up to 10 µm (PM_10_), given that the resulting particle is larger and less dense a respiratory droplet, which could increase the time it remains in the atmosphere. However, other research also carried out in Italy suggests the opposite in terms of the possible transmission of the virus via material particles [39]. Other studies carried out in Spain on days with an increase in PM from Saharan dust support this last hypothesis [40].

The lags in which associations were established with the different disease indicators in our analysis, both for PM_10_ and for NO_2,_ are compatible with both the needed incubation times of the virus (of between 2 and 12 days [20]) and the different processes of worsening of the disease [22]. In all cases a logical offset was observed in the lags from the time of case detection to death in the case of PM_10_. The association with p-value below 0.05 observed in the short term (lags 0 and 5) for NO_2_ with the incidence rate, hospital admission rate and ICU admission rate, is noteworthy. We understand that such a short-term association cannot be justified by the damage of NO_2_ to the immunological system in the short-term and supporting infection mechanisms, as has been reported, but rather by the worsening of prior respiratory and cardiovascular pathologies. The concentrations of NO_2_ in Madrid are related to both an increase in mortality due to circulatory causes [41, 42] and respiratory causes [41, 42], as well as to hospital admissions [43], especially for people over age 75 [32].

The two-variable models that include PM_10_ and NO_2_ together with the dependent variables related to COVID-19 analyzed show more robust associations for NO_2_ than for PM_10_ in all cases. Prior studies carried out in Madrid related to the impact of both PM_10_ [44] and to NO_2_ [42] on all-cause daily mortality, show greater RR for NO_2_ RR: 1.012 (95% CI 1.010, 1.014) compared to PM_10_ RR: 1.009 (95% CI 1.006, 1011). While these RR slightly overlap, this greater risks of NO_2_ could account for the fact that NO_2_ shows a more robust association in the two-variable models that combine both primary pollutants with a high collinearity (in the period analyzed the correlation between both pollutants is 0.519 with *p* < 0.0001) (Table 2).

The results of the models of COVID-19 rates concerning the association of the average daily concentrations between 0 and 14 days prior (shown in Table 4) show behavior that is similar to what was described earlier for daily values. It should be noted that the short-term association of NO_2_ disappeared, which is in accordance with the hypothesis of the acute effect of exacerbation of circulatory and respiratory symptoms described for the daily values. Furthermore, in general, the lags in which associations were established for NO_2_ and for PM_10_ were more long-term, which is compatible with less acute effects. It should be noted that in this case an association did appear between PM_10_, NO_2_ and mortality due to COVID-19.

### The association of the meteorological variables (temperature and absolute humidity)

Maximum daily temperature (Tmax) showed more robust behavior compared to the variables related to COVID-19 rates in the modeling process than average and minimum daily temperatures, thus it was selected as the variable for the analysis. There was a greater number of associations with p-values below 0.05 and greater statistical significance. The finding that Tmax was more closely related to COVID-19 rates than Tmin may be counter-intuitive. One explanation could be that Tmin is usually recorded around 7 a.m., a time when very little human activity occurs outdoors, while Tmax is usually recorded at around 4 p.m. [45].

The increasing trend in maximum daily temperature and the increasing trend in absolute daily humidity (AH) shown in Fig. 2b, c is coherent with climate conditions that are usually present in Madrid during the period analyzed [46].

The results shown in Table 3 indicate the existence of an association with p-value below 0.05 for both Tmax and AH. The relationship was negative for all of the all indicators analyzed, except for mortality, for which there was no detected association. That is to say, low and humid temperatures are related to higher incidence rates. In vitro studies have shown that SARS-CoV is inactivated at both higher temperatures and humidities [47], the results founded are in line.

On the other hand, the serological study of the prevalence of SARS-CoV-2 in Spain (ENE-COVID) [23] indicates that a lower prevalence of COVID-19 in Spain was produced in coastal regions that, during the time of the study and in general, are characterized by higher temperatures and humidity than the interior areas of the Peninsula [47].

Seasonal respiratory viruses are transmitted through aerosols, large respiratory droplets, or by direct contact with fomites [2]. Lower temperatures could also be an important factor that favors the diffusion of the SARS-CoV-2 in temperate regions [13, 46]; in the same way, relatively low humidity could also contribute to greater transmission of the new virus [48]. Other studies show results that are similar to what we found in our analysis, in terms of humidity and the incidence of COVID-19 transmission [49–52].

On the other hand, the results of our study show that higher temperatures correlates with lower incidence and severity of the disease. This may be compatible with a protective association of the temperature. Similar results have been found in other studies carried out in different parts of the world, including China [6, 53], the United States [54] and Spain [15], even though the evidence of the role of temperature on the incidence of the virus is still unclear [54]. The results of some studies are contradictory to what we describe here [55], and still other studies carried out in different parts of the U.S. are inconclusive.

Extreme temperatures can also affect morbidity and mortality due to different causes [56, 57]. However, the temperatures registered during the study period are far from cold spells or heat wave temperatures [56] for the city of Madrid. Thus the expected association of temperature would be to facilitate or make more difficult the transmission of the virus. This result agrees with the lags in which the associations were found, shown in Table 3 for daily values and in Table 4 for 14-day averages. It can be observed that in neither of the two cases are there short-term lags (lag 0), but there are lags in the values similar to the incubation period of the virus [20] and with the worsening of the disease [22].

The association of AH is predominant in the two-variable models compared to Tmax, both for daily values as well as 2-week averages. This result agrees with what was found in a study of eight U.S. cities [54] which concluded, “Humidity was observed as the best predictor for the coronavirus outbreak followed by temperature“.

### Results of the all variables models (pollution and meteorology)

In general, the behavior of the models with all variables was similar to that of the two-variable models (Table 4), both in terms of daily values and of average values, with a predominant association with concentrations of NO_2_ over PM_10_ and of AH over Tmax.

The higher RR values obtained for the 14-day average values as compared to daily values (Table 5), especially for NO_2_, show that it is not the higher daily levels of pollution that most correlated the incidence and severity of the disease; rather it is the average values. This supports that the preventive public health measures related to pollution and COVID-19 must be structural and aimed at decreasing the pollution in the city, as opposed to conjectural measures to avoid episodic situations.

The RR of these environmental factors are small (in terms of relative risk) and, by themselves, cannot explain the behavior of the incidence and severity of COVID-19, which is explained by social distancing and public health measures not considered in our analysis, this assumption is similar to the findings founded in the First report of the WMO COVID-19 Task team [58]. Only RR related to AH are relevant, but the low mean values corresponding to AH (as can been observed in Table 1), makes its contribution to the COVID-19 variables not so high.

### Strengths and limitations of this study

One of the principal strengths of this study is the longitude of the series used. Although the series was of 212 days, or 4 months, it is longer than the majority of studies carried out to date. The duration of the series allowed for carrying out generalized linear models with control variables such as trend, seasonality and the autoregressive component. In addition, it also allows for models with all the variables that include both meteorological and pollution-related variables, which is a significant improvement compared to the many investigations carried out to date in this field, that have used two variables correlations corresponding to series of 30 days.

Another strength of this study is the robust nature of the findings, first between the lags in which associations were established, which are coherent with biological mechanisms that link the different variables analyzed and the incidence and severity of the disease with the period of incubation and the course of the disease. This analysis was possible thanks to the daily data on COVID-19 variables as opposed to accumulated data or data averaged over time.

In addition, not only was a single association found between an indicator and the disease, rather there were four indicators with coherent results between them. This robustness extends as well to the relationship between the two variables and models with all variables and to the results obtained for the daily as well as averaged series. On the other hand, not only daily values are used but also averages of 0–14 days, which eliminates the weekly seasonality that exists in the dependent and independent variables. The longitude of the series is also, paradoxically, a weakness. Only a 4-month period was considered, without accounting for the complete annual variation.

About the study design, the analysis is a descriptive observational study. Specifically, it is a population-based ecological study. Generally, in epidemiological studies it constitutes a level of basic evidence. This type of study does not allow a causal relationship; but it constitutes useful exploratory approach [59]. Another limitation of the study consist in the lock-down period, this period was completely anomalous in terms of the decrease in air pollutants levels. This determines people exposure, which was different from usual situation. For example, in the analysis conducted, the impact of ozone concentrations on health was not included, due to previous analysis performed in Madrid city [60] have concluded that the threshold for ozone values from which effects on health are detected is over 60 µg/m^3^ (daily average). This value was not reached any day in the study period.

On the other hand, the conditions under which the data were obtained correspond to a period in which the declaration of cases only occurred when people already presented important symptoms of the disease.

The study conducted by the authors corresponds to an ecological time series design, with all the epidemiological limitations inherent in this type of study, especially the ecological fallacy. Both of the aforementioned points show the need for prudence in extrapolating the results to other situations in time other than those corresponding to the time this study was carried out. Nevertheless, this study could be evaluated with other methodologies that could complement the analysis carried out, a methodology such as propensity score matching [61], even a cohort study could help to improve the quality of observed findings. However, due to the immediacy of the COVID-19, there has not been enough time to carry out a follow-up study to guarantee better scientific evidence. On the other hand, the time series analysis methodology used has been previously implemented in Spain, for example, studying the relationship between COVID-19 and environmental variables such as traffic noise [62] and analyzing the effect of particulate matter from Sahara dust in the incidence of COVID-19 [40]. In addition, there are other examples using time series design to analyze the association between COVID-19 and air pollution carried out in Italy [63], France [64], United Kingdom [65], China [66], and Latin America [67]. Finally, ecological studies are a very efficient tool for making decisions in public health at the short-term [68] and very useful in the context of the current pandemic to identify environmental risks factors. Later studies will carry out a more in-depth and joint analysis of the impact of climate variability, air pollution and other factors that are extrinsic to the transmission of COVID-19.

## Conclusions

The results of this study indicate that the environmental variables analyzed are related to the incidence and severity of COVID-19 in the Community of Madrid. In general, low temperatures and low humidity in the atmosphere are associated with increased spread of the virus. Air pollution, especially NO2_,_ is associated with a higher incidence and severity of the disease. The 14-day average values imply a greater risk than daily values.

However, the RR of these environmental factors are small and by themselves cannot explain the behavior of the incidence and severity of COVID-19, which is explained by social distancing and public health measures not considered in our analysis.

## Data Availability

It’s an ecological analysis so the study does not involve human subjects. The data in relation to COVID-19 used in this study are subject to statistical secrecy and, therefore, are not freely available.

## Abbreviations

COVID: Coronavirus disease
ICU: Intensive Care Unit
INE: National Statistics Institute
AEMET: State Meteorological Agency (AEMET)
MITECO: Ministry of Ecological Transition and Demographic Change
CCF: Cross-correlation functions
RR: Relative Risk
